# Offshore wind farm avoidance by a discard‐feeding seabird is independent of local fishing activity

**DOI:** 10.1111/1365-2656.70279

**Published:** 2026-06-08

**Authors:** Rosemarie Kentie, Rob S. A. van Bemmelen, Judy Shamoun‐Baranes, Eric W. M. Stienen, Ruben C. Fijn

**Affiliations:** ^1^ Department of Coastal Systems Royal Netherlands Institute for Sea Research (NIOZ) Den Burg Texel the Netherlands; ^2^ Department of Bird Ecology Waardenburg Ecology Culemborg the Netherlands; ^3^ Institute for Biodiversity and Ecosystem Dynamics University of Amsterdam Amsterdam the Netherlands; ^4^ Department Birdlab Research Institute for Nature and Forest (INBO) Brussels Belgium

**Keywords:** fisheries, GPS tracking, individual variation, Lesser Black‐backed Gull, macro‐avoidance, OWF, seabirds, step selection analysis

## Abstract

1. Offshore wind farms are increasing rapidly in number and scale, presenting potential risks for seabirds. Some seabirds actively avoid offshore wind farms, whereas others are attracted to them. Both avoidance and attraction can have detrimental effects, including habitat loss and increased collision mortality. Because commercial fishing is typically restricted within wind farm boundaries, apparent avoidance by discard‐feeding species may reflect attraction to nearby fishing activities rather than actual wind farm avoidance
2. Here we studied the temporal variation in avoidance and attraction of 58 GPS‐tracked Lesser Black‐backed Gulls (*Larus fuscus*) to both the offshore wind farm and fishing activity within the foraging range from their breeding colony. If apparent avoidance of offshore wind farms was caused by an attraction to nearby fisheries, we expected a lower selection strength of the offshore wind farm during weekdays when fishing activity is much higher compared to weekends.
3. We used a step selection analysis to estimate relative selection strength including individual variability and tested if the selection strength for wind farms and fishing activity differed between the pre‐breeding, breeding and post‐breeding periods, between sexes, and between weekdays and weekends.
4. At the population level, gulls avoided the offshore wind farm during most periods and selected areas with fishing activity compared to areas without. The selection strength of wind farms and attraction to fisheries varied substantially between individuals, with several males being attracted to the wind farm during the breeding and post‐breeding period. Variation between individuals in avoidance of offshore wind farms varied most during the breeding period. The attraction to fishing activity did not differ between periods and sex. Fishing intensity was four times lower during the weekends than during weekdays. However, we found no support that gulls differed in wind farm avoidance between weekdays and weekends, suggesting that avoidance is not primarily driven by fishing exclusion within wind farms.
5. This research contributes valuable empirical data to improve the biological realism of Collision Risk Models by demonstrating that individual variability in avoidance behaviour warrants the inclusion of behavioural heterogeneity in these models rather than assuming uniform population responses.

## Graphical abstract

Fishing is not allowed within wind farms; therefore, discard‐feeding seabirds may appear to avoid wind farm areas. We found that most Lesser Black‐backed Gulls avoided the wind farm area, but not each individual in each period. Avoidance of the wind farm was not driven by fishing exclusion within its perimeter.

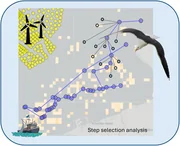

## 1 INTRODUCTION

Coastal shelf seas, such as the North Sea, are among the most heavily human‐impacted marine habitats in the world (Halpern et al., 2015). Fishery intensities and shipping traffic are high, and offshore wind farms are increasing in number, area and the size of individual turbines (Emeis et al., 2015; Gusatu et al., 2021). To become carbon neutral in 2050, the EU's wind power action plan will accelerate construction of new offshore wind farms in the near future (European Commission, Directorate‐General for Energy, 2023). These human activities can impact wildlife, especially apex predators such as seabirds (Furness & Camphuysen, 1997; Hazen et al., 2019).

The presence of offshore wind farms can negatively affect seabirds directly through increased mortality rates due to collisions with wind turbines, or indirectly via habitat loss if seabirds avoid wind farm areas (Mendel et al., 2019). Offshore wind farms can also act as a barrier, leading to increased flight costs (Thaxter et al., 2024). An important driver of these negative effects is the extent to which birds are either attracted to or avoiding wind farms (Dierschke et al., 2016; Johnston et al., 2022). For example, some species such as Great Cormorants (*Phalacrocorax carbo*) and European Shags (*Phalacrocorax aristotelis*) have been observed using the base of turbines to perch (Dierschke et al., 2016), while in contrast, other species such as Divers (*Gavia* spp.) and Northern Gannet (*Morus bassanus*) avoid offshore wind farms, thereby reducing their available foraging area (Mendel et al., 2019; Peschko et al., 2021).

Because fishing is prohibited in many offshore wind farms, discard‐feeding seabirds such as Lesser Black‐backed Gull (*Larus fuscus*), Northern Fulmar (*Fulmarus glacialis*) and Black‐legged Kittiwake (*Rissa tridactyla*) (Sherley et al., 2019) may have a lower tendency to enter wind farms. Perceived macro‐avoidance of wind farms could therefore be driven by the attraction to fishing vessels outside and the absence of fishing vessels within them, rather than avoidance of wind turbines themselves. On the other hand, fish abundance and biomass can be locally higher within wind farms than in nearby surroundings (Reubens et al., 2013), which may attract seabirds. As experiments have investigated the feasibility of passive fishing (gillnets, handline fishing, mechanical jigging and multi‐species pots) within wind farms (Neitzel et al., 2024), this may become more common at a wider scale in the future, which may attract seabirds to wind farms and therefore potentially increase collisions risk.

Due to flight characteristics and nocturnal foraging, large gulls such as Lesser Black‐backed Gull and Herring Gull (*Larus argentatus*) are considered highly vulnerable to collisions (Bradbury et al., 2014; Furness et al., 2013). However, studies show mixed results for wind farm avoidance of Lesser Black‐backed Gulls (Dierschke et al., 2016; Johnston et al., 2022; Vanermen et al., 2014). This variation may reflect individual differences in foraging strategies (Camphuysen et al., 2015; Isaksson et al., 2016), or environmental responses, as seen in Black‐legged Kittiwakes (O'Hanlon et al., 2024). Although the potential effects of the exclusion of fishing activities from wind farms on avoidance have been acknowledged (Dierschke et al., 2016; Vanermen et al., 2019), fishing activity in the vicinity of wind farms was not included in previous studies. With the development of platforms such as the Global Fishing Watch (www.globalfishingwatch.org), it is now possible to account for fishing vessel presence when studying avian response to wind farms.

Lesser Black‐backed Gulls have a varied diet ranging from marine to terrestrial food sources (Camphuysen et al., 2024). Previous studies have shown that Lesser Black‐backed Gulls spend less time at sea during the weekends when fishing activity is lowest compared to weekdays (Sotillo et al., 2019; Tyson et al., 2015). Foraging patterns of gulls can also vary between sex, with males foraging mostly on fisheries discards at offshore trawlers and females more on land and nearshore (Camphuysen et al., 2015). Other factors such as distance to foraging areas likely also play an important role in habitat choice. Lesser Black‐backed Gulls breeding on Texel, the Netherlands, for instance, spent between 25% and 78% of their time when they were not in the colony at sea (depending on sex and breeding phase) (Camphuysen et al., 2015), while gulls breeding in other colonies hardly spent any time offshore (Clewley et al., 2023; Gyimesi et al., 2016; Spelt et al., 2019).

Developments of GPS‐tracking now allow studying wind farm avoidance behaviour in greater detail (Fijn et al., 2022; Johnston et al., 2022; van Bemmelen et al., 2023) and at the individual level (O'Hanlon et al., 2024). Here, we studied at‐sea habitat selection of Lesser Black‐backed Gulls from a colony located relatively close to an offshore wind farm (~40 km). We first investigated how much time they spent at sea and inside the perimeter of the offshore wind farm. Then we studied simultaneously the attraction to or avoidance of wind farms and fishing activity using step selection analyses (SSAs; Thurfjell et al., 2014; Avgar et al., 2016; Chatterjee et al., 2024). SSA is based on the principles of resource selection analysis and compares the properties of actual used steps (two subsequent GPS positions) with randomly chosen available steps at each time interval. Recent developments in these models made it possible to add individual random slopes (Muff et al., 2020), and thereby investigate individual differences in habitat selection.

The main aim of this study was to investigate whether attraction to fishing activities influences the apparent selection of offshore wind farms by gulls. We hypothesized that gulls showed a lower selection for wind farms during weekdays when fishing activity is much higher compared to weekends. If males in this colony spent more time at sea than females (see e.g. Camphuysen et al., 2015), then we would expect a stronger effect in males than females. To address the potential interaction between fishery activity and wind farm selection, we had four specific objectives: (1) estimate daily patterns in fishing intensity around the colony and the time spent at sea by gulls; (2) quantify selection strength for areas with fishing activity and the nearest offshore wind farm; (3) test whether gulls show differential selection for wind farms between weekdays (high fishing activity) and weekends (low fishing activity); and (4) examine how these patterns vary across breeding periods (pre‐breeding, breeding and post‐breeding), between sexes, and among individuals.

## 2 MATERIALS AND METHODS

### 2.1 Data collection

**FIGURE 1:**
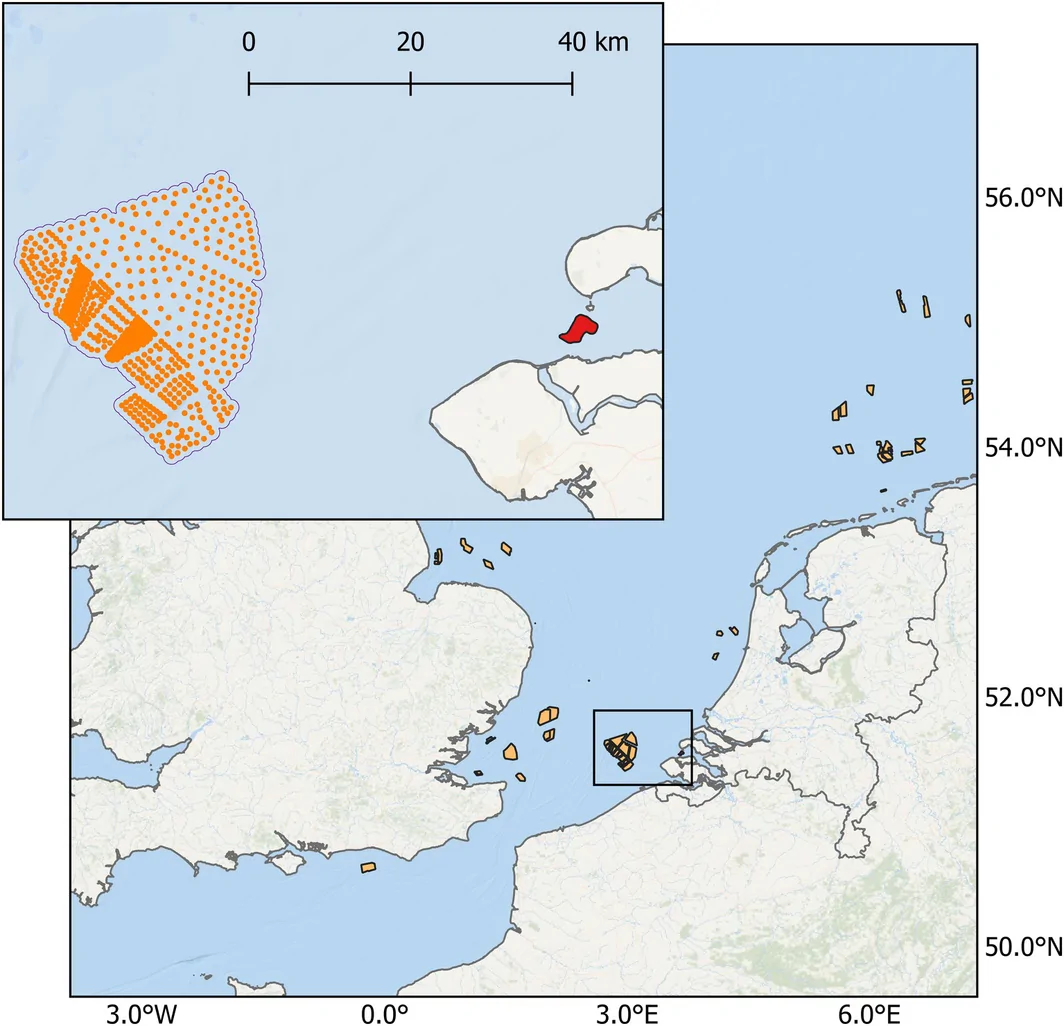
Map of the research area in the southern North Sea. Offshore wind farms in production during the study period are in orange, with the individual turbines and the perimeter around them shown in the inset. The breeding colony at Neeltje Jans (51°37′45″ N 3°41′28″ E) is shown in red.

Lesser Black‐backed Gulls breeding on the artificial island Neeltje Jans (Figure 1) in the south of the Netherlands were tagged with GPS trackers (OT‐15‐3GCT) of 15 g manufactured by Ornitela (Vilnius, Lithuania; ornitela.com), attached with a permanent wing harness (Thaxter et al., 2015). Gulls were captured with walk‐in cages on the nest during the incubation phase and additionally received a metal ring and a coloured plastic ring with a unique inscription. Individuals were weighed and measured (wing, head‐bill, bill and tarsus), and sex was based on head length (Camphuysen et al., 2024). Lesser Black‐backed Gulls are dimorphic, with a high accuracy (95%) of sexing adults based on biometry (Camphuysen, 2013). 25 Gulls were tagged between 20‐5‐2020 and 2‐6‐2020, and 50 gulls were tagged between 25‐5‐2021 and 2‐6‐2021. All tags were less than 3% of the body weight. To limit data gaps through low battery charge, the settings of the GPS trackers varied per time of day, location, season and with battery voltage. The highest resolution was 20 s inside the offshore wind farm area within the gull's expected home range; the lowest resolution was 30 min inside the colony and between nautical dusk to civil dawn. Outside the breeding period, before May and after July, the resolution was set to 20 min regardless of time of day. See Figure S1 in the Supporting Information for location of geofences and example tracks. A subset of nests was monitored in 2020 (20 nests) and 2021 (50 nests). Based on egg counts and hatching dates, we estimated that laying date of the first egg was on average on 13 May in both years.

### 2.2 Ethical statement and permits

Fieldwork was carried out under permits issued by Natuurmonumenten (permit WB.262.01.1904221.18) and het Zeeuws Landschap (permit number WC/AR120048). Tracking of lesser black‐backed gulls was performed under the project licence for animal procedures AVD401002015102 of the Central Authority for Scientific Procedures on Animals (CCD) and individual ringing permits granted by the Dutch Centre for Avian Migration and Demography (Vogeltrekstation).

### 2.3 Habitat data

**FIGURE 2:**
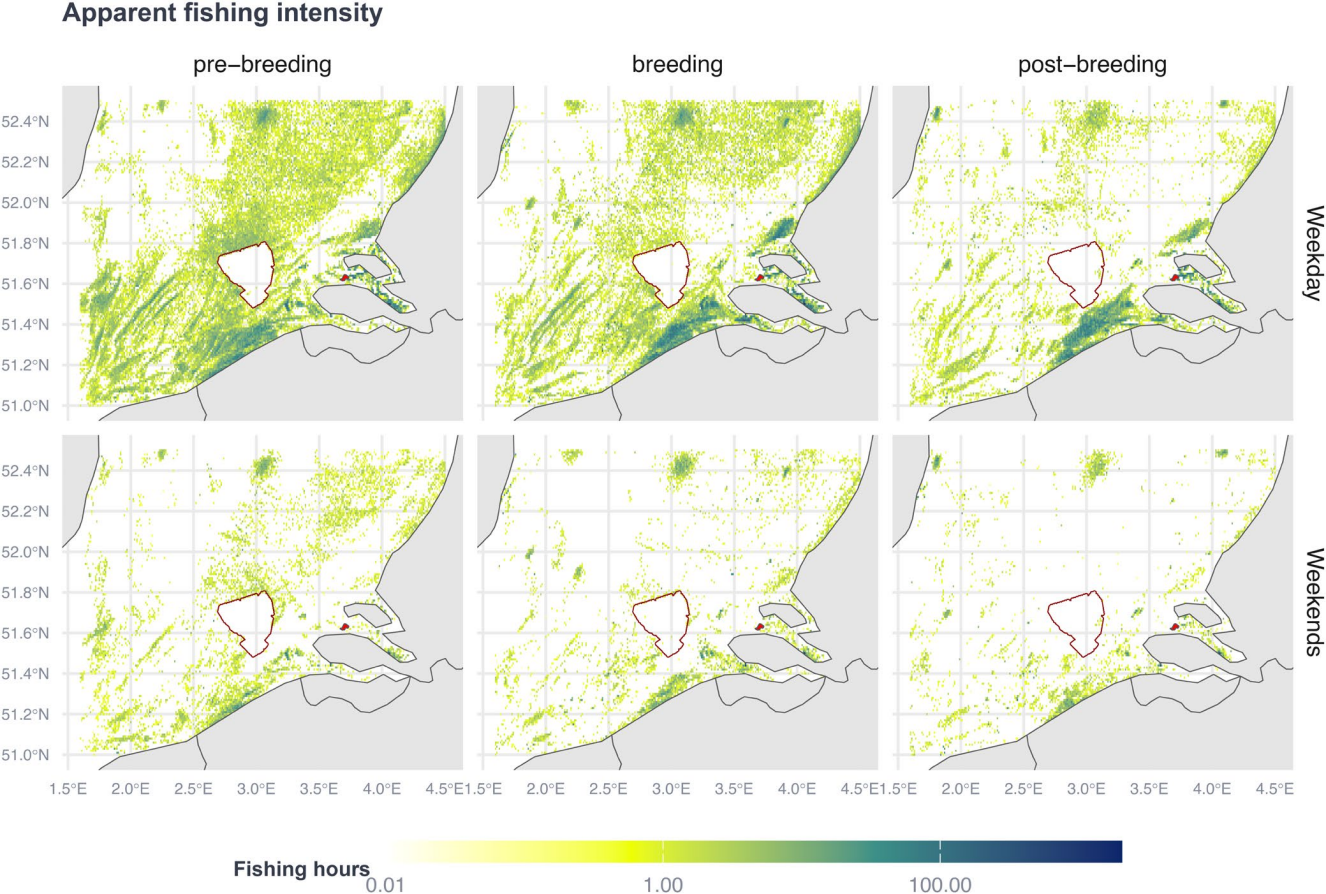
Apparent fishing intensity in hours per grid cell (0.01° × 0.01°) per breeding period separated by weekday (Monday–Thursday) and weekends (Friday–Sunday), summed for 2021 and 2022. The perimeter of the offshore wind farm within the expected foraging range is shown in large red polygon, and the breeding colony in the smaller red polygon.

The offshore wind farm cluster nearest to the breeding colony lies approximately 40 km in a westerly direction (Figure 1) and consists of the wind farms in Dutch waters (Borssele I‐V) and Belgian waters (Norther, C‐Power, Rentel, Northwind, Seastar, Belwind, Northwester 2 and Mermaid) (Figure S1). The wind turbine locations from Borssele were downloaded from ESRI NL (published by Nationaal Georegister), and those from the Belgian wind farms were downloaded from GeoNetwork (published by the Royal Belgian Institute for Natural Sciences). The specifics for each wind farm can be found in Table S1. For each turbine, we calculated a buffer of 1 km around its base to draw a polygon for the entire wind farm cluster. Fisheries data were downloaded from Global Fishing Watch (www.globalfishingwatch.org), which provides apparent fishing intensity (in hours per day) based on AIS and sailing speed, using version 3 of their AIS data pipeline (Figure 2). We used a resolution of fishing intensity data of 0.01° on a daily scale (from midnight‐to‐midnight UTC).

### 2.4 GPS‐data selection

We selected data of GPS‐tagged Lesser Black‐backed Gulls between 01‐03‐2021 and 31‐09‐2022 and kept only those points in the North Sea (north of 51.02° N). Most gulls arrive in the breeding area in April and start to migrate southwards in August (Camphuysen et al., 2024). We removed GPS location data which were obtained by four or less satellites to assure accuracy of the GPS positions (0.195% of all data locations). We checked the remaining positions for abnormal speeds and removed positions where the trajectory speed (distance divided by time) calculated between locations was >75 km/h but the instantaneous GPS speed registered by the loggers was <25 km/h (0.055% of the locations). Visual inspection of the data showed that these positions were very likely erroneous. We iterated this procedure three times. We categorized three seasonal periods based on the nest data from 2020 and 2021: the pre‐breeding period between first arrival in the North Sea area until 15 May, the breeding period including egg‐laying, incubation and chick care between 15 May and 15 July, and the post‐breeding period from 15 July until departure from the study area. We did not include whether a bird was actively breeding since we lacked this information in 2021 for individuals tagged in 2020.

### 2.5 Time spent at sea and in the offshore wind farm

We estimated the time in hours per day a bird spent at sea, within the perimeter (with a 1 km buffer) of the offshore wind farm and in an area with fishing activity, by summing the time intervals between two GPS fixes and using the second GPS fix to define habitat. Because of the differences between GPS fix intervals between areas and periods, we resampled tracking data to 20‐min intervals (the minimum recoding interval of GPS locations in the study period) with a tolerance of 5 min using the function track_resample from the amt package (Signer et al., 2019). We analysed if time spent at sea, in wind farms or in areas with fishing activity, differed between breeding periods and between weekdays (Monday–Thursday) or weekends (Friday–Sunday) and sex in separate models. We used generalized linear mixed models with a tweedie distribution, as the response data had a cluster of zeros but no negative values, using the package glmmTMB (Brooks et al., 2017). We included an interaction between breeding period and weekend, breeding period and sex and weekend and sex, as well as random intercepts for individuals. Model fit was confirmed with the package Dharma (Hartig, 2022), and models with and without the interactions were compared using AIC. Of the best models, we estimated the marginal means with the package emmeans (Lenth, 2024).

### 2.6 Step selection analysis

SSA estimates relative selection strength by comparing habitat characteristics of used steps to randomly generated alternative steps, where positive values indicate attraction and negative values indicate avoidance. We applied SSA to estimate selection strength for areas with fishing activity and offshore wind farms. Steps are defined as the connections between consecutive individual GPS locations. Step length is the distance between consecutive locations, and turning angle is the angle between consecutive steps (Fortin et al., 2005; Signer et al., 2019). The randomly chosen available steps are generated for each used step using the distribution of step lengths and turning angles from the data, allowing a comparison between actual and potential movement choices. A used step with the generated random steps is treated as a cluster, therefore reducing the effects of temporal autocorrelation (Avgar et al., 2016). Time intervals between GPS locations should be regular, not too short so that individuals can move between habitats, and not too long to capture behavioural responses to habitat features (Florko et al., 2025; Thurfjell et al., 2014). We resampled the tracking data to 20‐min intervals with a tolerance of 3 min, which is stricter than for the analyses for time spent per day, using the function track_resample from the amt package (Signer et al., 2019), keeping bursts (consecutive locations at 20‐min intervals) of more than three locations. This interval allowed gulls flying at an average commuting ground speed of 32.4 km/h (Shamoun‐Baranes et al., 2016; Thaxter et al., 2019, comparable to our findings) to cover approximately 10 km per step—sufficient to move between different habitat types while maintaining adequate resolution to detect responses to wind farms and fishing vessels.

We generated 25 random steps for each used step using the random_steps function from the package amt (Signer et al., 2019), using a gamma distribution for step lengths and a Von Mises distribution for turning angles. See Figure S2 for the distribution of step lengths and turning angles. We removed randomly generated positions which fell outside the study area and those on land. For each end point of the step, we extracted whether gulls were within the offshore wind farm perimeter, or within a grid cell where fishing activity took place during that day. To be able to estimate if the day during the week influenced the movement of individuals, we defined if a step started during weekdays (Monday–Thursday) or the weekend (Friday–Sunday).

To estimate the selection strength for the offshore wind farm and areas with fishing activity, we ran SSA models as conditional logistic regressions using mixed models with a Poisson distribution with a glmmTMB in R (Brooks et al., 2017; Muff et al., 2020). Used steps versus random steps were included as a dependent variable, and step ID (the grouping variable to link observed with random steps) as the random intercept of which we fixed its variance to 10^6^ to avoid shrinkage to the mean (see Muff et al., 2020). To estimate individual differences in selection strengths, we included individual random slopes per environmental variable. The natural log of step length and the cosines of the turning angle were included as fixed effects to reduce bias in parameter estimation (Forester et al., 2009). To reduce the number of models and computational demand, we used a staged process comparing AIC values in steps (Morin et al., 2020). We first tested whether the selection strength for the environmental variables ‘within wind farm’ and ‘at fishing activity’ differed per breeding period (pre‐breeding, breeding and post‐breeding) by comparing models with and without these interactions. Using the best supported model, we evaluated if selection strength for offshore wind farms and fishing activity differed per sex. Finally, we used the best supported model to test whether there was a difference in selection strength for ‘within wind farm’ between weekdays and weekends.

## 3 RESULTS

### 3.1 Fishing intensity and distribution

The spatial distributions of fishing intensity (Figure 2) show that almost no registered fishing took place within the offshore wind farm, and most fishing took place south‐west of the colony near shore. Daily fishing intensity was four times lower during the weekends (on average 270 h per day) than on weekdays (on average 1066 h per day) (Figure S3).

### 3.2 Distribution and time spent at sea, in the wind farm and in areas with fishing activity

**FIGURE 3:**
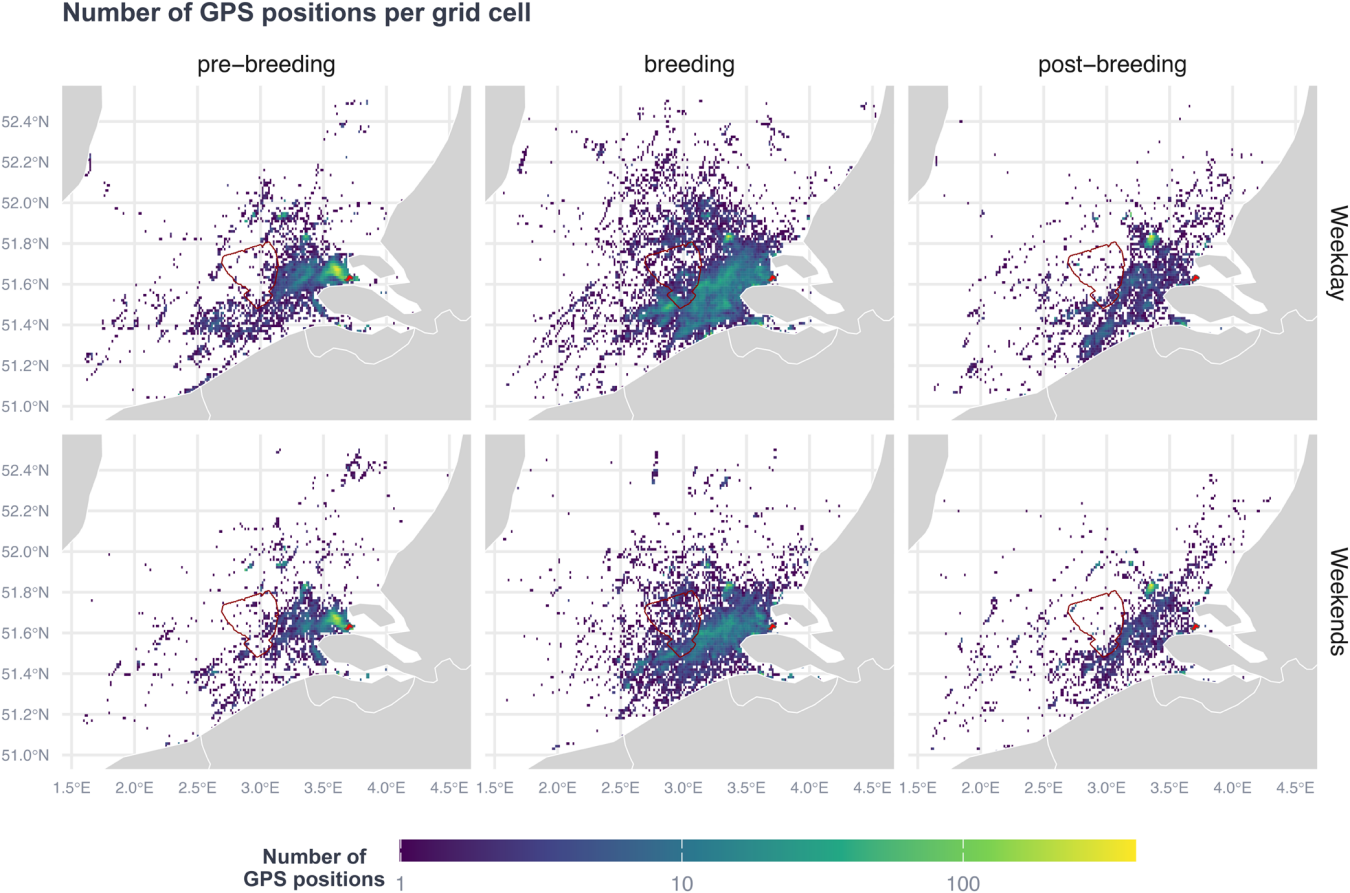
Density of resampled GPS locations of Lesser Black‐backed Gulls breeding at Neeltje Jans in the South of the Netherlands per breeding period divided by weekday (Monday–Thursday) and weekends (Friday–Sunday) per 1 × 1 km grid cells. The breeding colony is shown in red, the perimeter of the offshore wind farm is shown in dark red. The locations are resampled to a 20 min interval.

**FIGURE 4:**
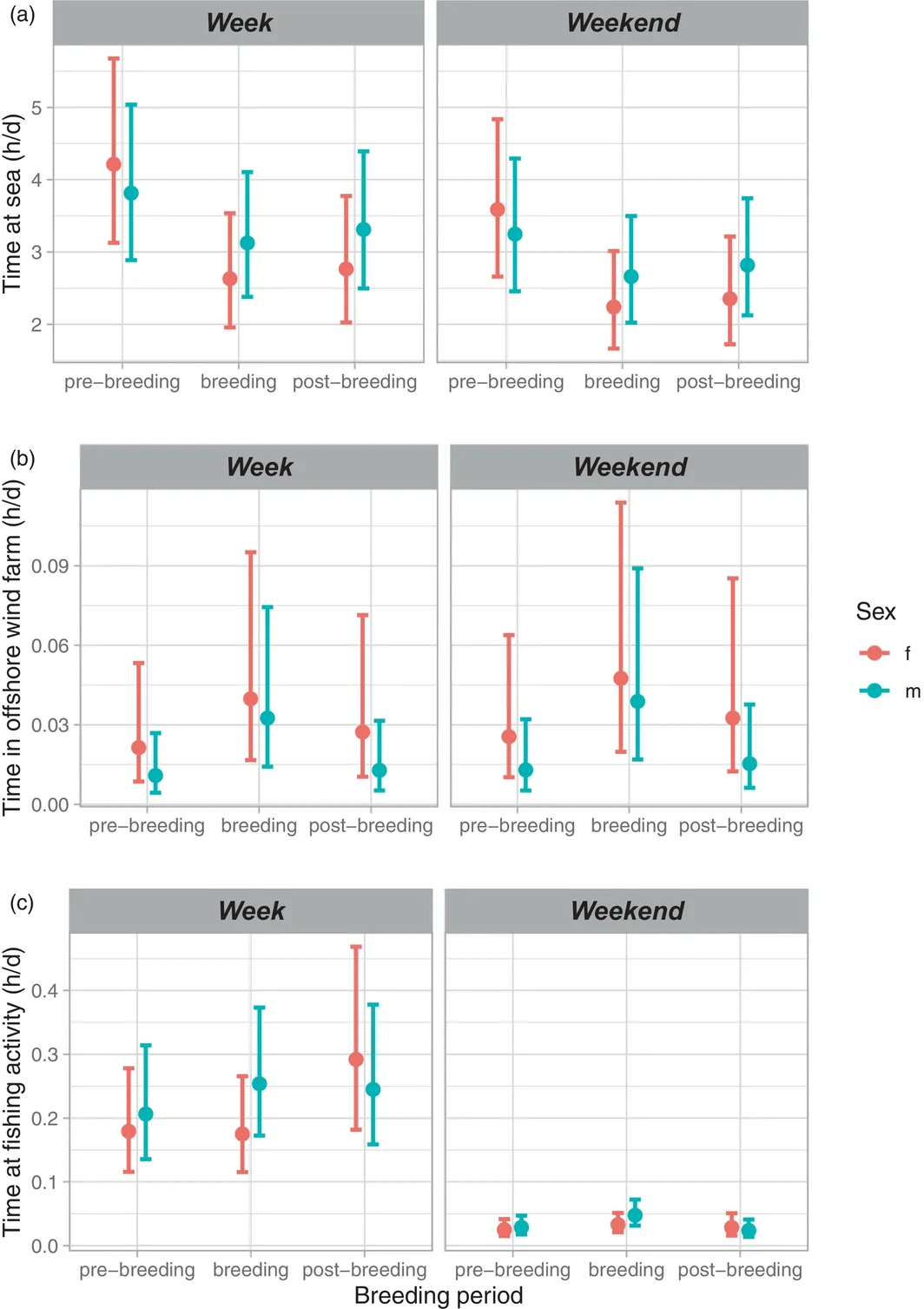
Time (in hours) spent per day (a) at sea, (b) in the offshore wind farm and (c) in area with fishing activity per breeding period, sex and during weekday (Monday–Thursday) or weekend (Friday–Sunday). Shown are the estimated marginal means of the top models. Note that the scale on the *y*‐axis differs.

At sea, Lesser Black‐backed Gulls primarily foraged west to south‐west of the colony between the wind farm and coastline (Figure 3). Gulls spent on average more time at sea during weekdays than weekends and during the pre‐breeding period (Table 1; Figure 4). Model estimates of time at sea ranged from 4.01 h/day (3.27–4.92 95% CI) on weekdays during pre‐breeding to 2.44 h/day (1.99–2.99 95% CI) on weekends during breeding. Males spent more time at sea than females during the breeding and post‐breeding period (Figure 4; Table 1; Table S2), but sexes did not differ in weekday/weekend patterns (Table S2). There was large variation in time spent at sea between individuals (Figure S4).

**TABLE 1:** Parameter estimates and statistics of top models on time spent per day at sea, in the offshore wind farm and at fishing activity per breeding period, weekend (Friday–Sunday) and weekdays (Monday–Thursday), year and sex, excluding non‐significant interactions. Individual ID was included as a random intercept.

Model estimates	Model: Time at sea			Model: Time in OWF			Model: Time at fishing activity		
Parameter	Estimate (SE)	*Z* value	*p*	Estimate (SE)	*Z* value	*p*	Estimate (SE)	*Z* value	*p*
Intercept	1.423 (0.154)	9.245	<0.001	−3.541 (0.474)	−7.465	<0.001	−1.437 (0.230)	−6.234	<0.001
Weekend	−0.161 (0.025)	−6.431	<0.001	0.177 (0.104)	1.699	0.090	−1.969 (0.166)	−11.850	<0.001
Breeding period									
Breeding	−0.471 (0.040)	−11.752	<0.001	0.620 (0.192)	3.221	0.001	−0.025 (0.120)	−0.206	0.836
Post‐breeding	−0.421 (0.064)	−6.596	<0.001	0.242 (0.285)	0.850	0.395	0.487 (0.165)	2.950	0.003
Sex (male)	−0.099 (0.207)	−0.480	0.389	−0.677 (0.643)	−1.054	0.292	0.140 (0.305)	0.459	0.646
Year (2022)	0.031 (0.030)	1.051	0.244	−0.604 (0.121)	−4.977	<0.001	−0.563 (0.073)	−7.733	<0.001
Breeding period × sex									
Breeding:male	0.272 (0.058)	4.667	<0.001	0.475 (0.299)	1.589	0.112	0.231 (0.158)	1.462	0.144
Post‐breeding:male	0.280 (0.083)	3.366	<0.001	−0.075 (0.400)	−0.186	0.852	−0.316 (0.214)	−1.476	0.140
Weekend × breeding period									
Weekend:breeding							0.299 (0.194)	1.537	0.124
Weekend:post‐breeding							−0.357 (0.265)	−1.347	0.178
Individual random effect									
Variance (SD)	0.594 (0.771)			4.613 (2.148)			1.079 (1.039)		

Gulls spent very little time in the wind farm cluster (Figure 4) and some individuals which spent time at sea never entered the perimeter (Figure S5). On average and including gulls that did not enter the wind farm, they spent more time in the wind farm during the breeding period (0.039 h/day; 2 min) than the pre‐ and post‐breeding period (Table 1; Figure 4). The top model based on AIC (Table S2) included the interaction between breeding period and sex; however, the parameter estimates including sex were not significant (Table 1). Interactions between weekend and sex or breeding period and weekend were not supported, nor was the difference in time spent in the offshore wind farm between weekdays and weekends significant (Table S2). While daily exposure was minimal, seasonal totals from arrival in the North Sea until departure averaged 12 h per gull in 2021 and 8 h per gull in 2022.

On average, gulls spent 0.22 h/day (13 min) in areas with active fishing activity during weekdays compared to 0.03 h/day (2 min) during the weekend (Figure 4; Figure S6), corresponding to the lower intensity of fishing activity during the weekend. The best model included the interaction between weekend and breeding period and between sex and breeding period; however, the parameter estimates including sex were not significant (Table 1). Gulls spent more time in areas with fishing activity in 2021 (0.29 h/day during weekdays, 0.22–0.39 95% CI) than in 2022 (0.18 h/day, 0.12–0.22 95% CI).

### 3.3 Attraction and avoidance of the offshore wind farm and areas with fishing activity

**FIGURE 5:**
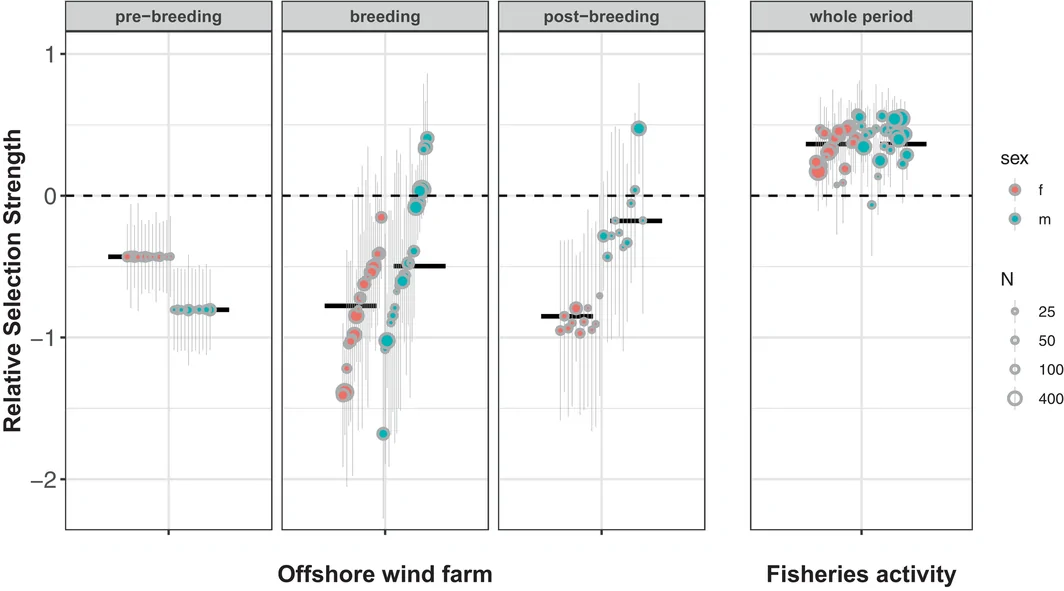
Conditional relative selection strength of Lesser Black‐backed Gulls for the offshore wind farm (OWF) during the pre‐breeding, breeding and post‐breeding period and areas with fishing activity, where positive values indicate attraction and negative values indicate avoidance. Colours show females and males, and within sex, individuals are ordered by the relative selection strength for the wind farm during the breeding period. The size of the dots represents the number of steps where at least one used or random end point fell within the wind farm, but only estimates of individuals with >10 of these steps are shown (considering that the selection strength can only be estimated if at least one used or random end point is within the wind farm). Black lines are population means per sex, grey vertical lines show individual confidence intervals. The log of step length was set at 6.57 km per 20 min and the cosines of the turning angle at 0, representing a bird in flight going straight.

Following GPS data resampling, the dataset for the SSA contained 98,785 steps from 59 individuals, generating 2,567,191 used and random steps. During the pre‐breeding period, fewer used and random steps ended in the wind farm than during the breeding and post‐breeding period (Table S3). On the population level, Lesser Black‐backed Gulls avoided offshore wind farms throughout the season, although the selection strength differed per breeding period (Table 2, Step 1) and sex (Table 2, Step 2; Figure 5). During the pre‐breeding period, males had a lower selection strength to wind farms than females. In contrast, during the breeding and post‐breeding periods, males showed a higher relative selection strength to wind farms than females (Table S4; Figure 5), and some males were attracted to the wind farm area (Figure 5). Gulls demonstrated strong attraction to areas with active fishing (Table S4; Figure 5), without any differences between breeding period nor strong support for a difference between males and females (Arnold, 2010) (Table 2). The magnitude of individual variation in selection strength in wind farm selection was greatest during the breeding period, for both females and males, while during the post‐breeding period, males showed higher individual variation than females (Figure 5). Figure S7 illustrates the differences habitat use of the individual with the lowest and highest selection strength to the offshore wind farm. Individuals also differed in the attraction to fishing activity, with some individuals showing indifference to fisheries (i.e. the confidence intervals crossed zero).

**TABLE 2:** Model selection results of the step selection analysis (SSA) using AIC to analyse if selection strengths for offshore wind farms (OWF) and areas with fishing activities differed per breeding period (Step 1), between females and males (Step 2), and between weekday and weekend (Step 3). Model estimates of the top‐ranking model results are shown in Table S4.

Step 1. Does selection strength differ per breeding period?					
Model + log(sl) + cos(ta) +	dLogLik	AIC	dAIC	df	Weight
OWF:period + fishing	16.6	2 204 183.3	0	10	0.944
OWF:period + fishing:period	17.8	2 204 188.9	5.6	14	0.056
OWF + fishing	0	2 204 208.5	25.2	6	<0.001
OWF + fishing:period	1.1	2 204 214.2	30.9	10	<0.001

Step 2. Does selection strength differ for sex?					
Model + log(sl) + cos(ta) +	dLogLik	AIC	dAIC	df	Weight
OWF:period:sex + fishing	5.1	2 204 179.1	0	13	0.485
OWF:period: sex + fishing:sex	5.9	2 204 179.5	0.4	14	0.404
OWF:period + fishing	0	2 204 183.3	4.1	10	0.061
OWF:period + fishing:sex	0.8	2 204 183.6	4.5	11	0.05

Step 3. Does selection strength differ between weekdays and weekend?					
Model + fishing + log(sl) + cos(ta) +	dLogLik	AIC	dAIC	df	Weight
OWF:period:sex	0	2 204 179.1	0	13	0.51
OWF:period:sex + OWF:period:weekend	2.9	2 204 179.2	0.1	16	0.49

Abbreviations: sl, step length; ta, turning angle.

### 3.4 Attraction to offshore wind farms during the weekend?

**FIGURE 6:**
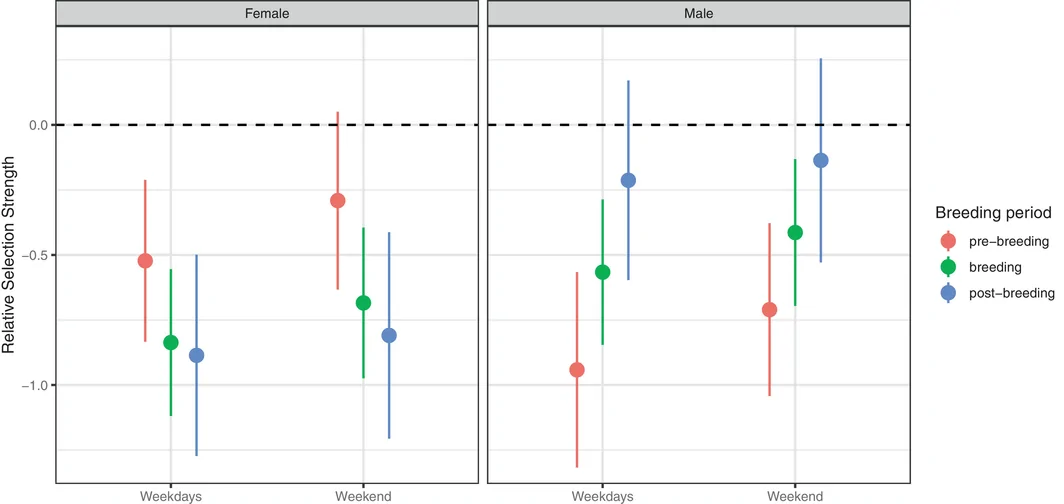
Conditional relative selection strength for the offshore wind farm during weekdays (Monday–Thursday) and weekends (Friday–Sunday) for females and males per breeding period, where negative values indicate avoidance. The log of step length was set at 6.57 km per 20 min and the cosines of the turning angle at 0, representing a bird in flight going straight.

We found no strong support for a difference in selection strength to offshore wind farms during weekdays and weekends in any breeding period (Table 2, Step 3; difference in AIC was 0.1 with three extra parameters for the model including weekend; Figure 6). Estimates show that gulls generally avoided offshore wind farms (negative selection strengths) during weekdays and at weekends, though the 95% CI overlapped with zero for females during the weekend in the pre‐breeding period and for males both during weekdays and the weekend during the post‐breeding stage.

## 4 DISCUSSION

We used a SSA to estimate Lesser Black‐backed Gulls' selection strength to areas with recent fishing activity and a large offshore wind farm cluster within the foraging range of their breeding colony. We found that, in general, gulls were attracted to areas with fishing activity and during most periods avoided the offshore wind farm. However, we observed substantial individual variation in these responses, particularly regarding wind farm avoidance during the breeding and post‐breeding periods. Gulls showing a neutral response or attraction to the offshore wind farm during the breeding and post‐breeding periods were mainly males. Gulls also spent more time in the wind farm during the breeding period compared to the pre‐ and post‐breeding periods. Importantly, we found no strong support that gulls differed in wind farm avoidance between weekdays and weekends, even though fishing intensity was four times lower. This suggests that avoidance of the wind farm is not primarily driven by fishing exclusion within its perimeter.

The strong population‐level attraction to fishing areas aligns with the species' known ecology as discard feeders (Camphuysen, 1995; Camphuysen et al., 2024; Sherley et al., 2019; Sotillo et al., 2014), particularly given that bottom trawling—the dominant fishing method around our study colony—produces abundant discards that serve as a key food source for seabirds (Sherley et al., 2019). However, this attraction does not necessarily indicate exclusive reliance on discards, as Lesser Black‐backed Gulls also actively forage on subsurface pelagic fishes and crabs (Baptist et al., 2019; Camphuysen et al., 2024). Although in our study, gulls showed strong selection for areas with active fisheries, the discrepancy between time spent at sea and time spent in areas with fishing activity, combined with their continued offshore foraging during weekends when fishing activity is minimal, suggests that gulls also engage in other foraging strategies and spend considerable time commuting and searching for resources. Lesser Black‐backed Gulls are known to specialize in their foraging behaviour (Camphuysen et al., 2024; Spelt et al., 2019), and the individual differences in selection strength to fisheries suggest some degree of specialization at sea. Previous studies found that males were more affiliated with offshore trawlers in the North Sea while females foraged more often on land (Camphuysen et al., 2015; Sotillo et al., 2019). Although we found that males spent more time at sea than females during both breeding and post‐breeding periods, we detected no clear patterns in time spent near fishing vessels between males and females, nor sex‐based differences in selection strength for areas with active fisheries. However, determining whether gulls actually foraged on discards requires more fine‐scale behavioural analyses.

While we found a macro‐avoidance of the offshore wind farm at the population level, Lesser Black‐backed Gulls have previously been shown to be weakly attracted to offshore wind farms (Dierschke et al., 2016). For instance, a study using ship‐based counts showed that gull numbers were higher in the offshore wind farm area post‐construction than pre‐construction (Vanermen et al., 2014), and GPS tracking revealed attraction to turbine foundations on the outer perimeter of the wind farm (Vanermen et al., 2019). Another study, which investigated avoidance and attraction of the offshore wind farm at several distance classes, found avoidance specifically in the 3–4 km zone but neither avoidance nor attraction when birds were closer (Johnston et al., 2022). Johnston et al. (2022) analysed trip‐level avoidance by comparing observed tracks with randomly rotated foraging tracks. In contrast, our SSA approach—similar to O'Hanlon et al. (2024) and van Bemmelen et al. (2023)—examined habitat selection at individual movement steps, quantifying selection probability at each decision point along the track. As gulls from different colonies often show different foraging patterns (Corman et al., 2016), and wind farm specifics such as turbine density may affect avoidance rates (van Bemmelen et al., 2023), responses to wind farm presence may also differ between colonies and wind farm layout. Despite our finding of population‐level avoidance during most periods, individual variation was evident, with some birds showing indifference or even attraction to the offshore wind farm as shown in previous studies (Dierschke et al., 2016; Johnston et al., 2022; Thaxter et al., 2018; Vanermen et al., 2014).

Individual variation in responses to the offshore wind farm was most pronounced during the breeding and post‐breeding periods, with some birds avoiding the area, others showing indifference, and a few individuals—mainly males—being attracted to the wind farm. A study on Black‐legged Kittiwakes also found substantial individual variation in selection strength for various environmental conditions, making it difficult to predict population level responses to potential local stressors such as offshore wind farms (O'Hanlon et al., 2024). Further research should investigate why some individuals are attracted to offshore wind farms, whether attraction represents a consistent behavioural trait that places some individuals at greater risk than others, and how these individuals behave within the wind farm environment (i.e. is the wind farm used as a foraging area or are individuals commuting through to other areas). Additionally, as our analysis included seasonal periods but not individual breeding status, future studies should examine whether attraction patterns vary with breeding stage, such as during chick provisioning when foraging demands and constraints differ. Beyond understanding avoidance patterns at the macro‐scale, examining within‐wind farm behaviour at the meso‐ and micro‐scale (Cook et al., 2018) is crucial for assessing individual collision risk with turbines.

We found no strong evidence that Lesser Black‐backed Gulls differ in selection pattern to offshore wind farms between weekdays and weekends. If gulls were not attracted to the wind farm primarily due to restricted fishery access, we would expect reduced avoidance during the weekend when fishing activity is minimal. While females showed slightly elevated selection strength during weekends in the pre‐breeding period compared to weekdays, and males appeared indifferent to the wind farm area during both weekdays and weekends in the post‐breeding period, the consistently negative selection strength suggests that the majority of gulls generally preferred to remain outside the wind farm regardless of nearby fishing activity. This macro‐avoidance of the offshore wind farm may result from direct turbine avoidance but could also be caused by differences in prey availability inside and outside the wind farm. Studies have documented higher fish abundance and biomass of pelagic species within offshore wind farms (Reubens et al., 2013), yet whether this increased abundance of prey within wind farms translates to enhanced foraging opportunities for gulls requires further investigation. Alternatively, gulls may exhibit regional site fidelity, returning to areas with fishing activity during weekdays. However, gulls are known to adapt their temporal foraging patterns to human schedules such as the timing of school breaks and opening and closing times of waste centres (Spelt et al., 2021), therefore, they are most likely also capable of anticipating the absence of fisheries during weekends.

Wind farm avoidance rate is an important parameter of Collision Risk Models, to which these models are highly sensitive (Cook et al., 2025; Masden et al., 2021). Our findings of temporal and sex‐related individual variation in avoidance behaviour have important implications for the use of Collision Risk Models for Environmental Impact Assessments and mitigation strategies. Current Collision Risk models typically apply fixed avoidance rates (Cook et al., 2025), but our results suggest that dynamic, context‐dependent parameters may improve prediction accuracy. For example, incorporating breeding period, sex, and, if possible, individual behavioural variation could refine risk assessments and identify periods or locations where mitigation measures are most needed. Furthermore, if fishing activities within offshore wind farms increase in the future, Lesser Black‐backed Gulls and other discard‐feeding seabirds may face elevated collision risks due to increased time spent in the wind farm. To develop effective conservation strategies, future research should focus on understanding the mechanisms driving seabird attraction and avoidance behaviours to and within wind farms. Such mechanistic understanding is essential for developing adaptive management approaches that can minimize seabird mortality while supporting renewable energy development, also for sites where the collection of field data is challenging.

## AUTHOR CONTRIBUTIONS

Rosemarie Kentie, Rob S. A. van Bemmelen, Ruben C. Fijn and Eric W. M. Stienen conceived the ideas and designed methodology; Ruben C. Fijn, Rob S. A. van Bemmelen and Eric W. M. Stienen collected the data; Rosemarie Kentie and Rob S. A. van Bemmelen analysed the data; Rosemarie Kentie led the writing of the manuscript. All authors contributed critically to the concept and drafts and gave final approval for publication.

## CONFLICT OF INTEREST STATEMENT

The authors declare no conflicts of interest.

## Supporting information


**Figure S1.** The offshore wind farm cluster showing the names of the wind farms, the geofences and two example GPS tracks resampled to 20 min.
**Figure S2.** The log of step lengths (km) and turning angles (radians) of the resampled GPS tracking data.
**Figure S3.** Daily and seasonal patterns of fishing intensity in hours per day, from 1 March until 30 August and shown separately for 2021 and 2022.
**Figure S4.** Mean daily time spent at sea per breeding period differentiated for weekdays (Monday–Thursday) and weekend (Friday–Sunday) per individual.
**Figure S5.** Mean daily time spent in the offshore wind farm per breeding period differentiated for weekdays (Monday–Thursday) and weekend (Friday–Sunday).
**Figure S6.** Mean daily time spent in areas with fighting activity per breeding period differentiated for weekdays (Monday–Thursday) and weekend (Friday–Sunday).
**Figure S7.** Density of resampled GPS locations during the breeding period of (a) the individual with the lowest selection strength to the offshore wind farm, and the (b) individual with the highest selection strength to the offshore wind farm.
**Table S1.** Specifications per wind farm project from the offshore wind farm cluster within foraging reach of the study colony of Lesser Black‐backed Gulls.
**Table S2.** Selection of models explaining time spent at sea, time spent in the offshore wind farm and time spent in areas with fishing activity, using AIC criteria.
**Table S3.** Sample sizes as used in the step selection analyses of individuals and GPS positions (used steps), individuals entering the offshore wind farm, and the number of individuals attracted to, were indifferent, or avoided the offshore wind farm based on whether or not the 95% CI crossed 0.
**Table S4.** Parameter estimates of the top‐ranking SSA model.

## Data Availability

All data is part of DELTATRACK and is freely available on Movebank (www.movebank.nl and Zenodo https://zenodo.org/records/15696782; Stienen et al., 2025).
