# Supplementary material for: Offshore wind farm avoidance by a discard‐feeding seabird is independent of local fishing activity
Source: J Anim Ecol. 2026 Jun 8;95(9):1727–40. doi: 10.1111/1365-2656.70279 (PMC13536243; doi:10.1111/1365-2656.70279)
Supplement: Supplementary file 1 [file JANE-95-1727-s001.pdf]

Supporting Information

Offshore wind farm avoidance by a discard-feeding seabird is independent of local fishing activity

Rosemarie Kentie[1], Rob S.A. van Bemmelen[2], Judy Shamoun-Baranes[3], Eric W.M. Stienen[4], Ruben C. Fijn[2]

1. Royal Netherlands Institute for Sea Research (NIOZ), P.O. Box 59, 1790 AB Den Burg, Texel, The Netherlands
2. Waardenburg Ecology, Varkensmarkt 9, 4101 CK Culemborg, The Netherlands
3. Institute for Biodiversity and Ecosystem Dynamics, University of Amsterdam, P.O. Box 94240, 1090 GE Amsterdam, The Netherlands
4. Research Institute for Nature and Forest (INBO), Havenlaan 88 bus 73, 1000 Brussels, Belgium

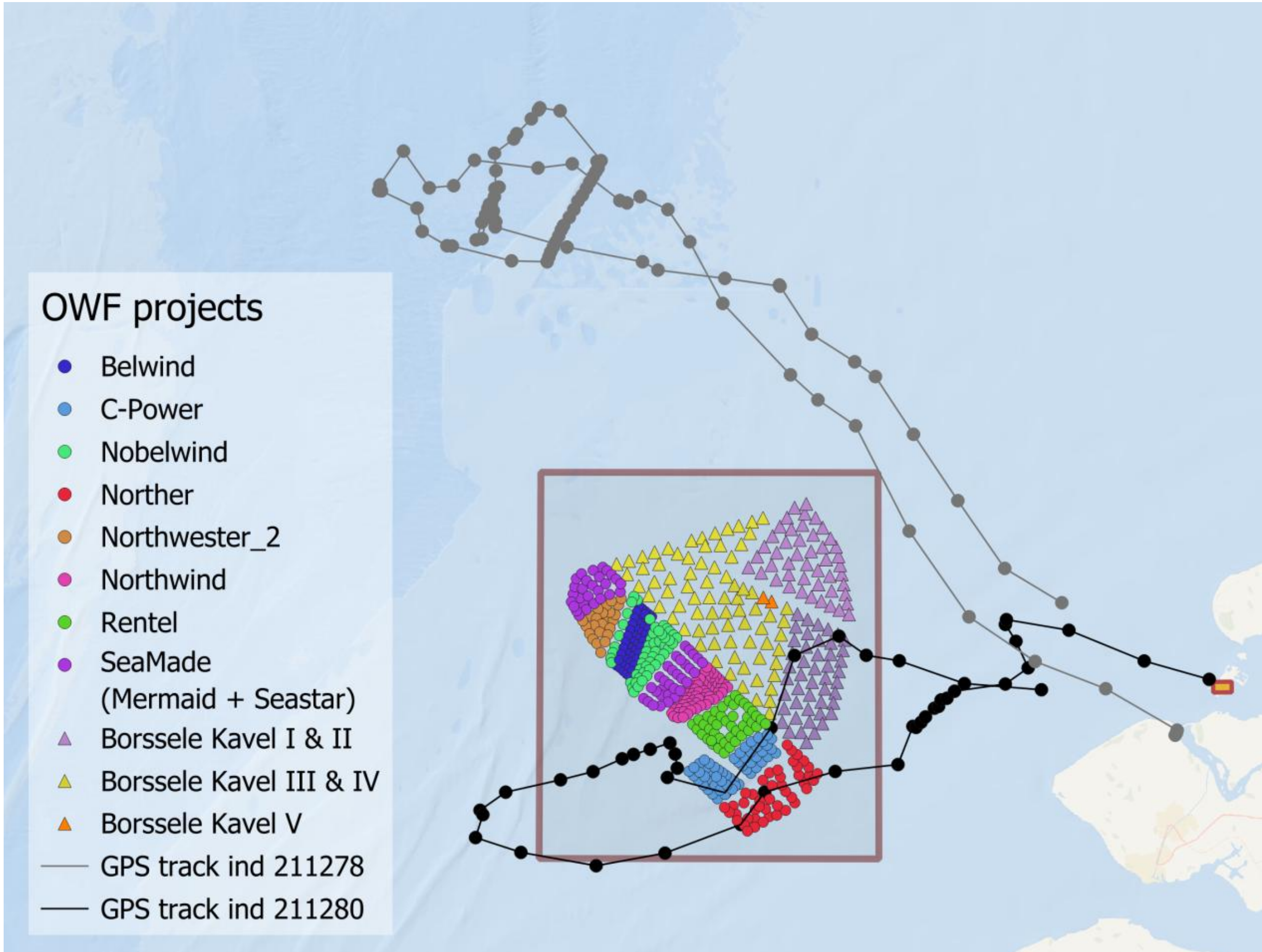

Figure S1. The offshore wind farm cluster showing the names of the wind farms, the geofences (around the wind farm and around the colony), and two example GPS tracks resampled to 20 min. Table S1 provides specifications per wind farm.

Table S1. Specifications per wind farm project from the offshore wind farm cluster within foraging reach of the study colony of Lesser Black-backed Gulls. The latest projects that became operational did so in February 2021, before GPS data selection.

Wind farm project	Hub Height (m)	Rotor Diameter (m)	Tip Height (m)	Year of First Operation	Number of Turbines
Belwind (Phase 1)	72	90	117	2010	55
Belwind (Haliade demo)	100	150	175	2013	1
Nobelwind	79	112	135	2017	50
Northwester 2	105	164	187	2020	23
SeaMade (Mermaid + Seastar)	109	167	192.5	2020	58
C-Power (Thornton Bank Phase 1)	95	126	158	2009	6
C-Power (Thornton Bank Phases 2 & 3)	95	126	158	2012	48
Northwind	71	112	127	2014	72
Borssele I & II	116.5	167	200	2020	94
Borssele III & IV	109	164	191	2021	77
Borssele V	105	164	187	2021	2

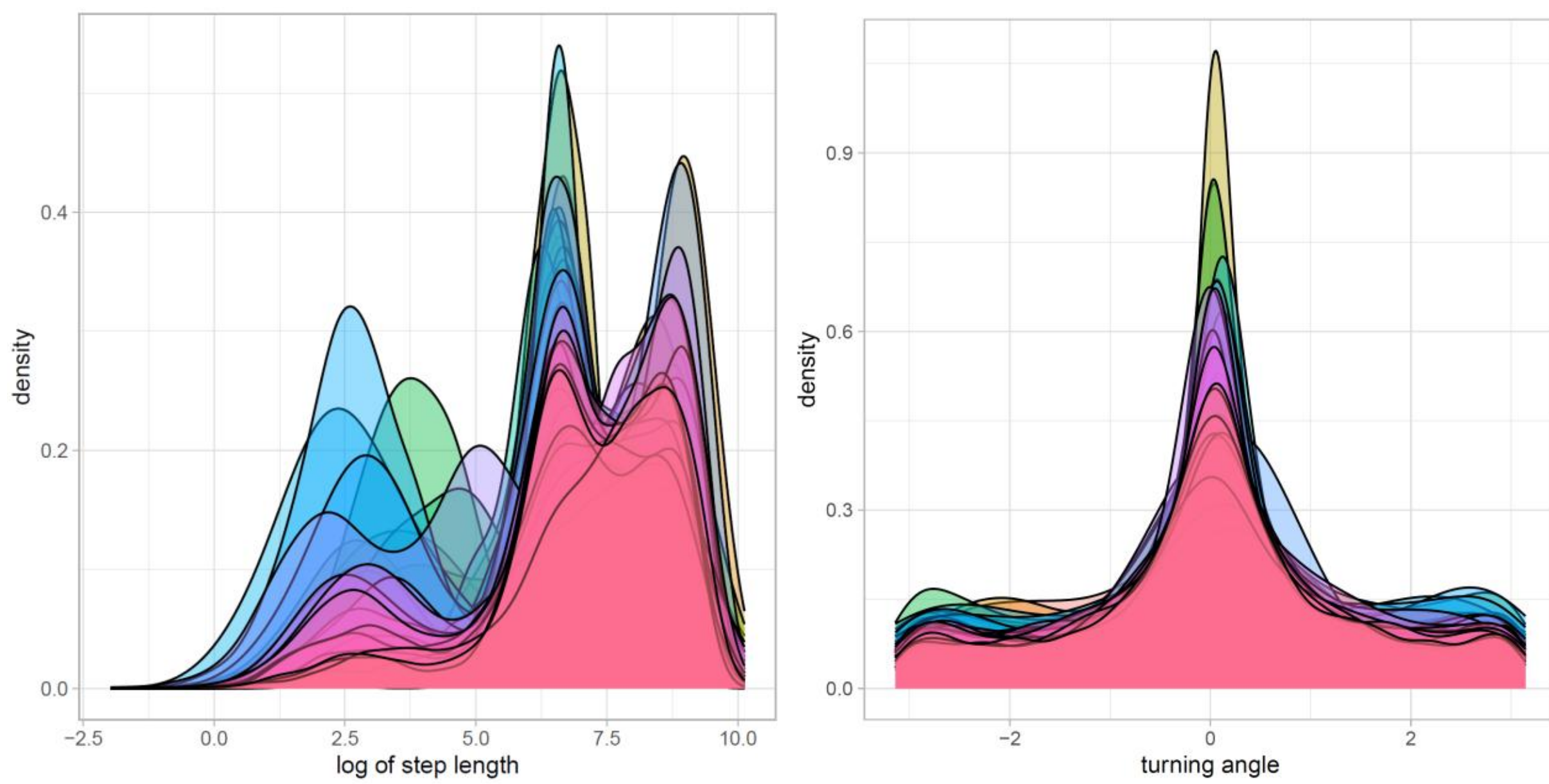

Figure S2. The log of step lengths (km) and turning angles (radians) of the resampled GPS tracking data. Individuals are coloured differently.

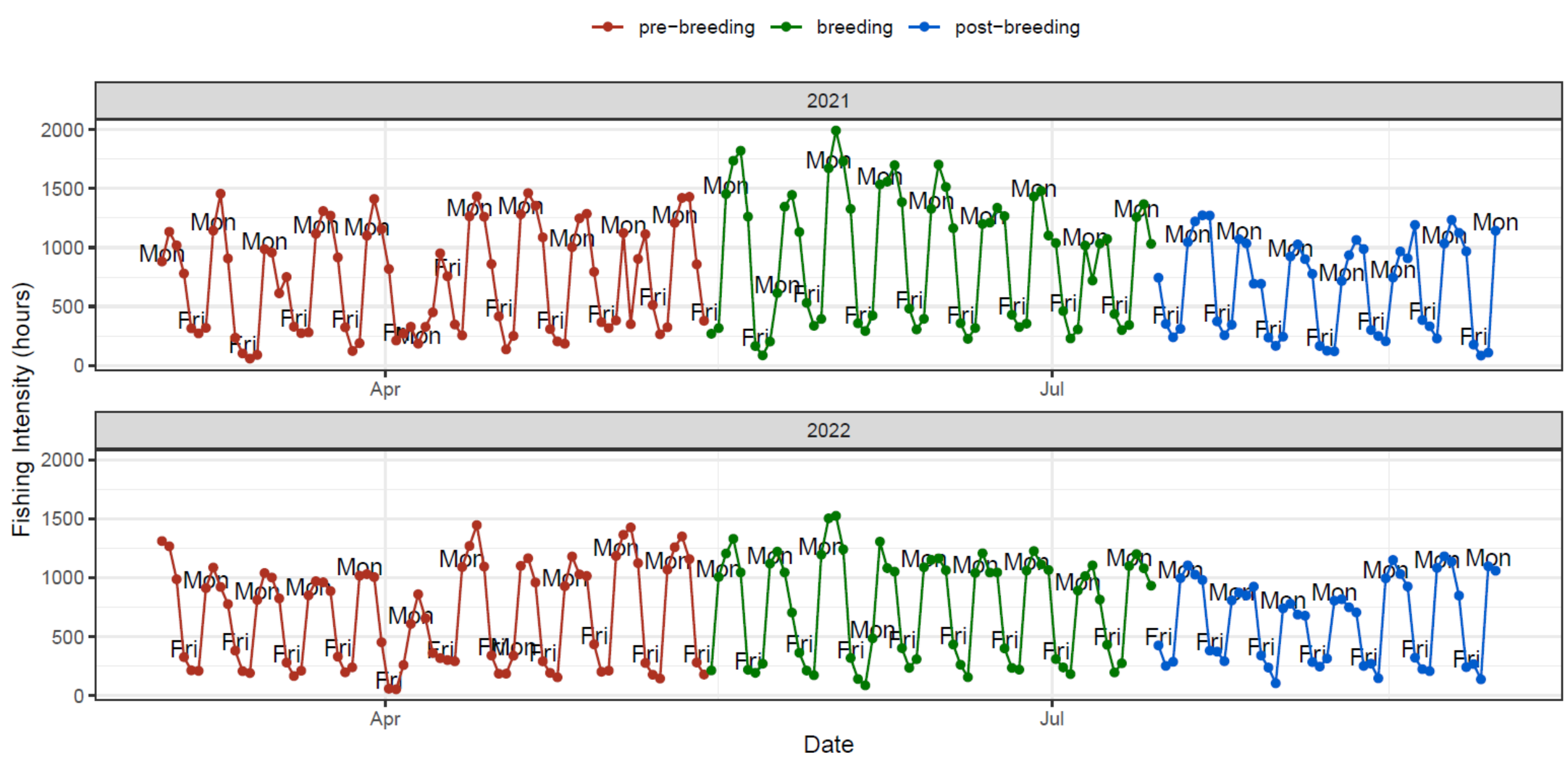

Figure S3. Daily and seasonal patterns of fishing intensity in hours per day, from 1 March until 30 August and shown separately for 2021 and 2022. Colours indicate the breeding period, Mondays and Fridays are marked with text.

Table S2. Selection of models explaining time spent at sea, time spent in the offshore wind farm and time spent in areas with fishing activity, using AIC criteria. Note that we only compared the interactions with our a priori null model (weekend + period + sex + year)

**Model time at sea**	**dLogLik**	**dAIC**	**df**	**weight**
weekend + period * sex + year	11.9	0.0	11	0.49
weekend * sex + period * sex + year	12.1	1.5	12	0.23
weekend * period + sex * period + year	12.8	2.1	13	0.18
weekend * period + weekend * sex + weekend * period + year	13.3	3.2	14	0.10
weekend + period + sex + year	0.0	19.7	9	<0.001
weekend * period + weekend * sex + year	1.3	23.0	12	<0.001
**Model time in offshore wind farm**	**dLogLik**	**dAIC**	**df**	**weight**
weekend + period * sex + year	2.3	0.0	11	0.40
weekend + period + sex + year	0.0	0.5	9	0.31
weekend * sex + period * sex + year	2.3	1.8	12	0.16
weekend * period + sex * period + year	2.6	3.4	13	0.08
weekend * period + weekend * sex + weekend * period + year	2.7	5.2	14	0.03
weekend * period + weekend * sex + year	0.4	5.8	12	0.02
**Model time in area with fishing activity**	**dLogLik**	**dAIC**	**df**	**weight**
weekend * period + sex * period + year	9.5	0.0	13	0.48
weekend * period + weekend * sex + weekend * period + year	10.3	0.3	14	0.41
weekend + period * sex + year	4.9	5.2	11	0.04
weekend * sex + period * sex + year	5.8	5.3	12	0.03
weekend * period + weekend * sex + year	5.8	5.4	12	0.03
weekend + period + sex + year	0.0	10.9	9	0.00

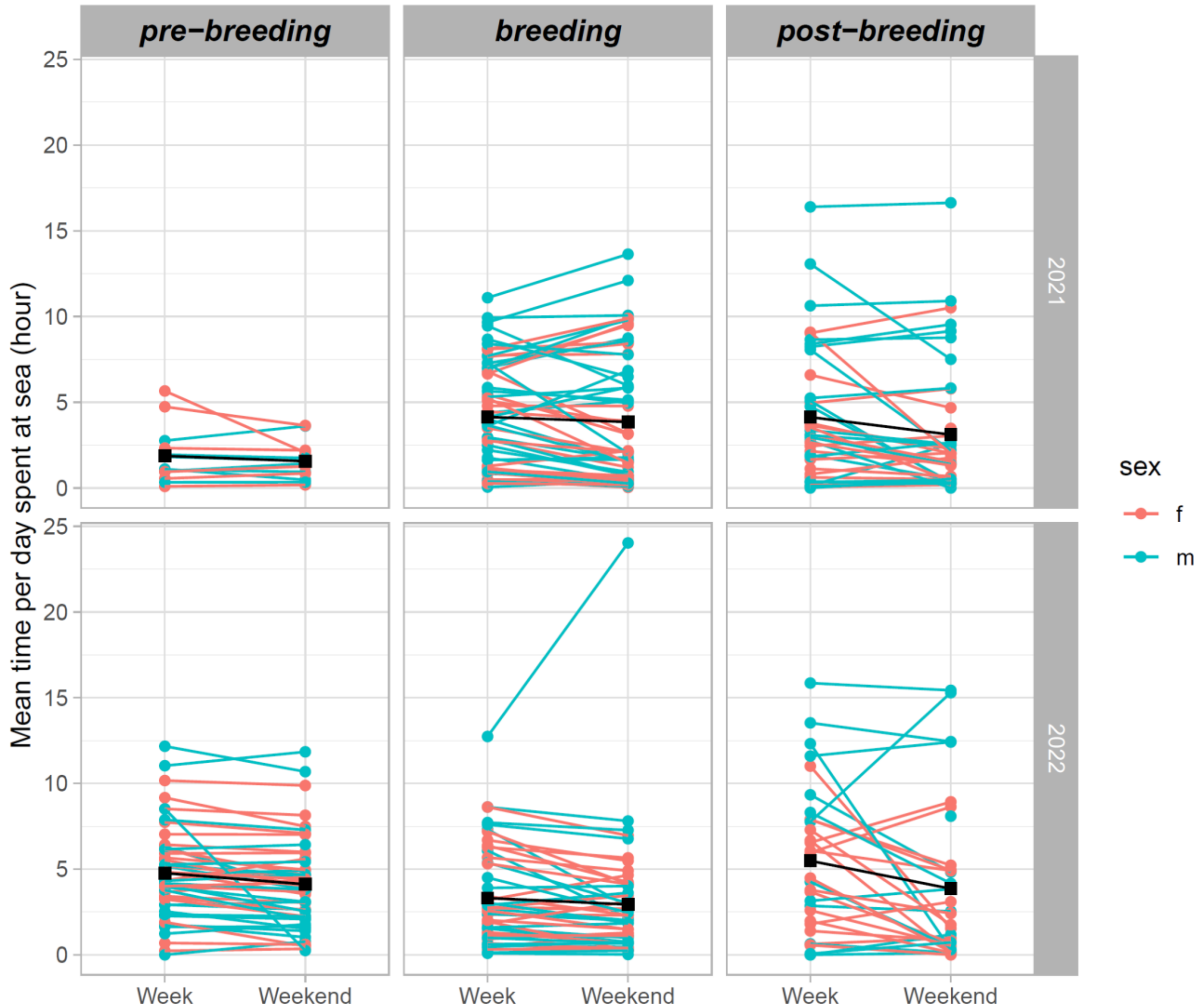

Figure S4. Mean daily time spent at sea per breeding period differentiated for weekdays (Monday – Thursday) and weekend (Friday – Sunday) per individual. Sexes are coloured differently, and the black squares and lines are the overall averages.

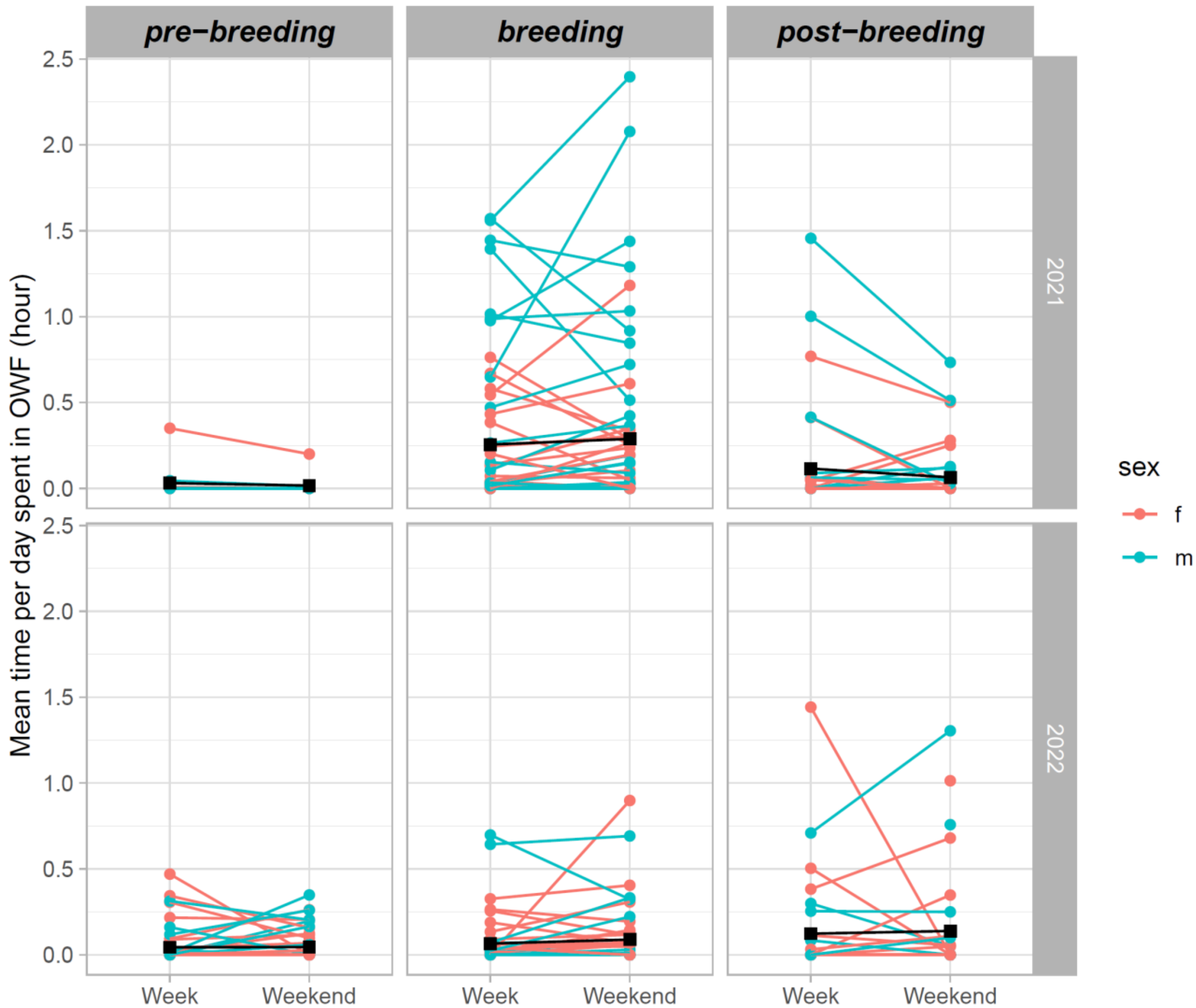

Figure S5. Mean daily time spent in the offshore wind farm per breeding period differentiated for weekdays (Monday – Thursday) and weekend (Friday – Sunday). Sexes are coloured differently, and the black squares and lines are the overall averages.

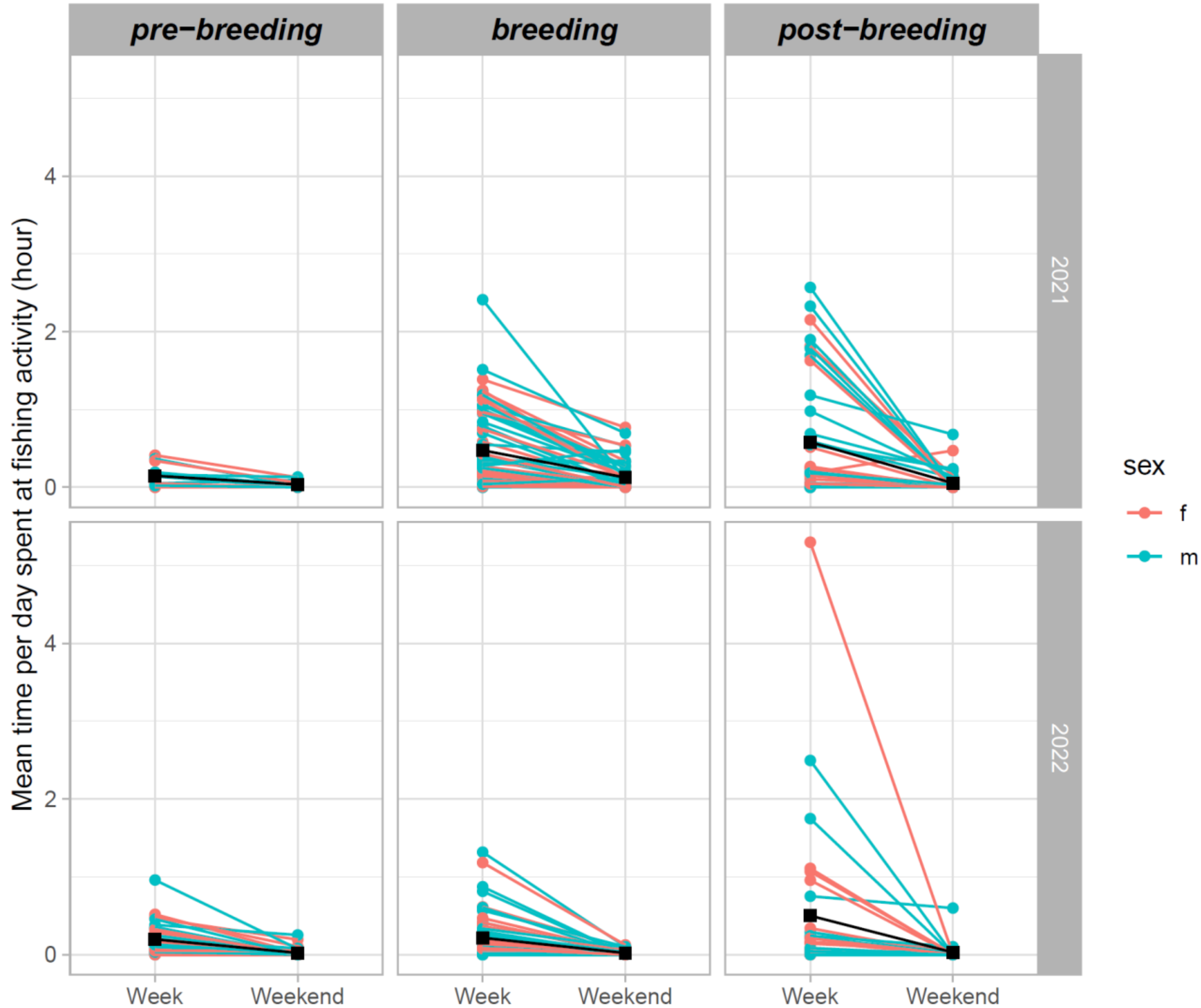

Figure S6. Mean daily time spent in areas with fighting activity per breeding period differentiated for weekdays (Monday – Thursday) and weekend (Friday – Sunday). Sexes are coloured differently, and the black squares and lines are the overall averages.

Table S3. Sample sizes as used in the step selection analyses of individuals and GPS positions (used steps), individuals entering the offshore wind farm, and the number of individuals attracted to, were indifferent, or avoided the offshore wind farm based on whether or not the 95% CI crossed 0. Note that this is sensitive to small sample sizes.

	**Pre-breeding period (< 15 May)**	**Breeding period (15 May – 15 July)**	**Post-breeding period (> 15 July)**
**Sample sizes**			
N individuals	52	58	46
N used steps	32013	48926	17810
N individuals in OWF	21	43	22
% of used endpoints in OWF	1.3 %	5.8 %	2.6 %
% of steps with at least one endpoint in OWF	5.6 %	20.4%	10.6 %
**N individuals***			
Attracted to OWF	0	1	1
Avoided OWF	24	23	10
Indifferent to OWF	0	21	11

* *Of individuals of which a random or used end location fell within the offshore wind farm for >10 steps*

Table S4. *Parameter estimates of the top ranking SSA-model. The negative estimates of the OWF parameters indicate avoidance of the offshore wind farm (OWF), positive estimates for fishing activity indicate attraction to areas with fishing activity during the three breeding periods.*

**Parameter**	**Estimate (SE)**	**z-value**	**P value**	**Random individual slope* (variance; st dev)**
In OWF pre-breeding period				0.0005; 0.0216
Female	-0.381 (0.117)	-3.274	< 0.001	
Male	-0.755 (0.144)	-5.24	< 0.001	
In OWF breeding period				0.279; 0.528
Female	-0.727 (0.140)	-5.206	< 0.001	
Male	-0.447 (0.137)	-3.358	0.001	
In OWF post-breeding period				0.106; 0.333
Female	-0.801 (0.181)	-4.414	< 0.001	
Male	-0.128 (0.180)	-0.712	0.476	
At fishing activity	0.414 (0.034)	12.161	< 0.0001	0.032; 0.180
log(step length)	-0.008 (0.001)	-5.033	< 0.0001	
cos(turning angle)	0.004 (0.005)	0.742	0.461	

* Not sex-dependent

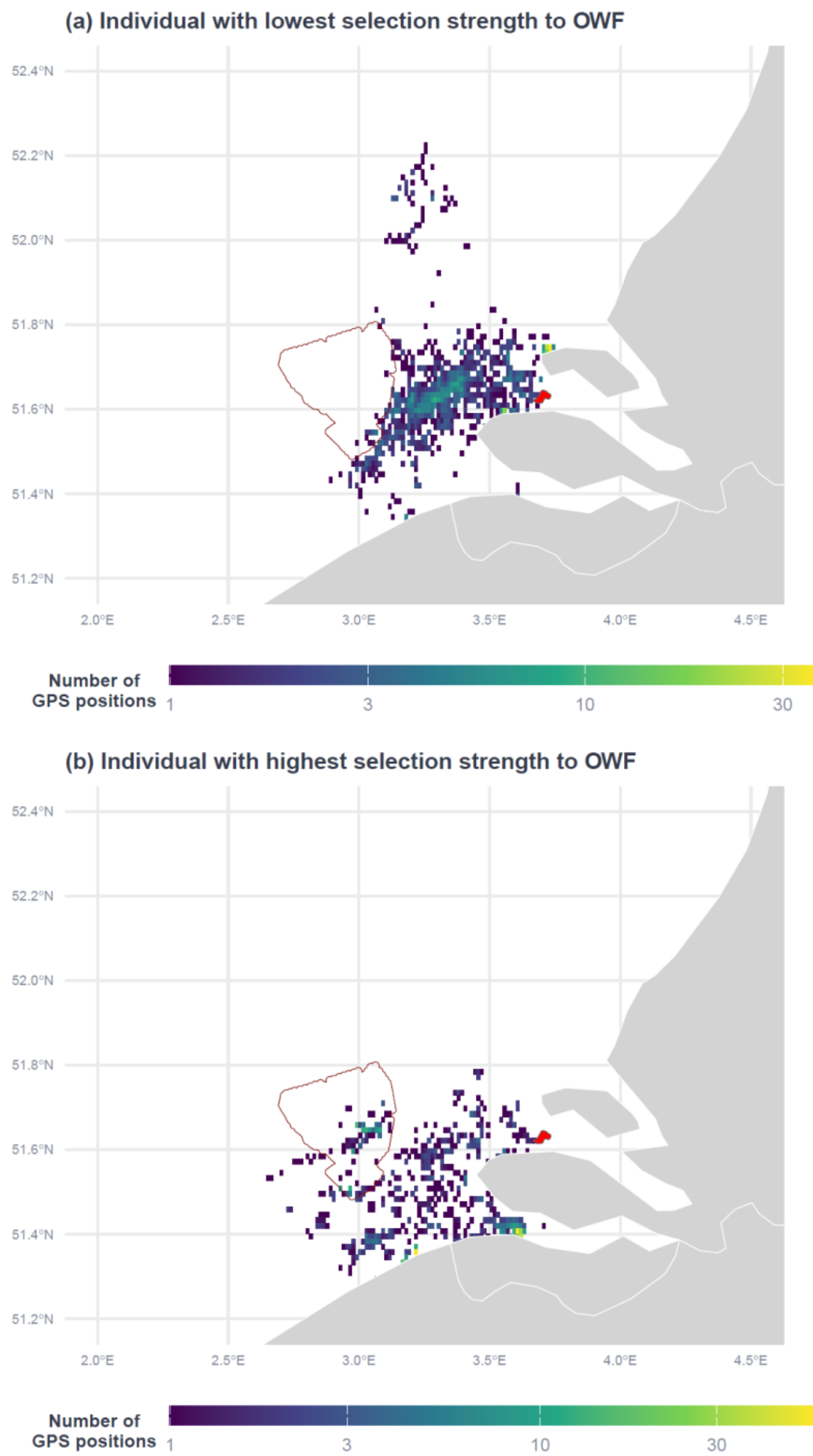

Figure S7. Density of GPS locations during the breeding period of (a) the individual with the lowest selection strength to the offshore wind farm, and the (b) individual with the highest selection strength to the offshore wind farm.
